# Supplementary material for: Quality Characteristics and Anthocyanin Profiles of Different Vitis amurensis Grape Cultivars and Hybrids from Chinese Germplasm
Source: Molecules. 2021 Nov 5;26(21):6696. doi: 10.3390/molecules26216696 (PMC8588336; doi:10.3390/molecules26216696)
Supplement: Supplementary file 1 [file molecules-26-06696-s001.zip › molecules-1393045-supplementary.pdf]

Table S1. General berry characteristics, total contents and antioxidant activities of phenolic compounds extracted from grape skins and seeds of the three groups during the 2017 and 2018 years

	Vitis amurensis		Iterspecific Hybrids		*V. vinifera*	
	Mean	SD	Mean	SD	Mean	SD
**General berry characteristics**						
Weight (g)	1.0182*	0.1981	1.5615	0.3326	1.7472	0.0120
Transverse diameter (mm)	11.7547*	0.8743	13.1616	0.9241	13.9332	0.3372
Longitudinal diameter (mm)	11.7869*#	0.8789	13.1711*	0.9489	14.1178#	0.0418
Total soluble solid (Brix)	13.40*#	2.34	16.84*	2.11	20.90#	0.23
Titratable acid (%)	1.53*#	0.41	1.12*	0.35	0.67#	0.01
pH	2.84#	0.17	3.01	0.17	3.60	0.55
**Total contents of phenolic compounds**						
Total phinols (TP, mg GAE/g DM)						
Skin	45.06#	8.48	32.30	9.55	37.89	6.53
Seed	37.77*	7.23	58.53*	26.00	125.47#	8.07
Total flavonoids (TFO, mg RAE/g DM)						
Skin	37.96#	7.50	24.54	7.26	28.29	4.90
Seed	34.39*	8.06	55.50*	27.07	123.33#	7.99
Total flavan-3-ols (TFA, mg CAE/g DM)						
Skin	2.26*	0.71	4.17*	2.51	12.72#	1.44
Seed	31.14*	7.59	50.79*	25.25	112.51#	5.66
Total anthocyanins	32.96*#	9.07	10.07	3.51	3.60	3.45
**Antioxidant activities of phenolic extracts** (μmol TE/g DW)						
DPPH free radical scavenging activities (DPPH)						
Skin	175.95	35.45	143.13	44.10	187.44	20.21
Seed	152.93*#	32.01	227.25*	86.38	414.59#	16.38
ABTS free radical scavenging activities (ABTS)						
Skin	158.92	30.92	131.69	36.99	173.92	18.00
Seed	132.30*	34.06	193.87*	80.20	375.10#	1.89
Ferric ion reducing antioxidant powers (FRAP)						
Skin	292.15	58.22	238.44	71.61	294.07	31.95
Seed	226.42*	49.93	331.38*	133.81	607.21#	10.97

* indicates the significant difference (p<0.05) vs. *V. vinifera* grapes for each indicator by Welch's ANOVA with Games-Howell. # indicates the significant difference (p<0.05) vs. interspecific hybrids.

Table S2. Correlation coefficients among total phenolic contents and antioxidant activities of phenolic compounds in grape skins

	TP	TFO	TFA	TA	DPPH	ABTS	FRAP
TP	1						
TFO	0.963**	1					
TFA	0.036	0.060	1				
TA	0.644**	0.779**	-0.500**	1			
DPPH	0.863**	0.772**	0.261*	0.361**	1		
ABTS	0.851**	0.752**	0.257*	0.338**	0.958**	1	
FRAP	0.896**	0.794**	0.197	0.398**	0.977**	0.953**	1

*, **: significance at $p<0.05$ and $p<0.01$, respectively. The abbreviations were the same as Table S1.

Table S3. Correlation coefficients among total phenolic contents and antioxidant activities of phenolic compounds in grape seeds

	TP	TFO	TFA	DPPH	ABTS	FRAP
TP	1					
TFO	0.998**	1				
TFA	0.997**	0.999**	1			
DPPH	0.975**	0.975**	0.978**	1		
ABTS	0.965**	0.968**	0.973**	0.991**	1	
FRAP	0.987**	0.988**	0.989**	0.979**	0.980**	1

*, **: significance at $p<0.05$ and $p<0.01$, respectively. The abbreviations were the same as Table S1.

Table S4. Correlation coefficients among the contents of simple diglucosides anthocyanins and antioxidant activities of phenolic extracts in grape skins

	Dp-3,5-diglc	Cy-3,5-diglc	Pt-3,5-diglc	Pn-3,5-diglc	Mv-3,5-diglc
DPPH	0.324**	0.299*	0.416**	0.391**	0.261*
ABTS	0.295*	0.281*	0.364**	0.387**	0.237
FRAP	0.368**	0.333**	0.466**	0.471**	0.262*

*, **: significance at $p<0.05$ and $p<0.01$, respectively. The abbreviations were the same as Table 2 and Table S1.
